# Supplementary material for: An open-label pilot study of recombinant granulocyte-colony stimulating factor in Friedreich’s ataxia
Source: Nat Commun. 2022 Aug 9;13:4655. doi: 10.1038/s41467-022-31450-w (PMC9363409; doi:10.1038/s41467-022-31450-w)
Supplement: Supplementary file 1 — Supplementary information [file 41467_2022_31450_MOESM1_ESM.pdf]

An open-label pilot study of recombinant granulocyte-colony stimulating factor in Friedreich's ataxia

Kemp et al.

Supplementary Information

Supplementary Figures

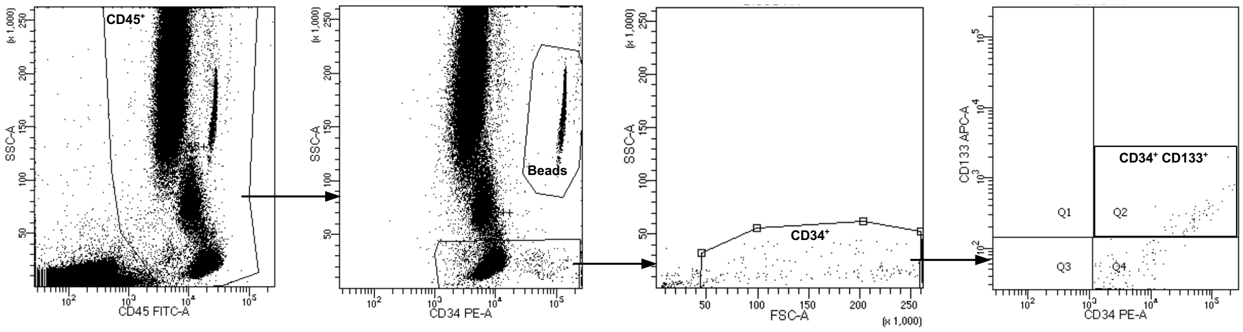

Supplementary Figure 1. Gating strategy to quantify peripheral blood hematopoietic stem cells. Flow cytometry gating strategy used to determine the number of $CD34^+$ and $CD34^+$ $CD133^+$ hematopoietic stem cells within peripheral whole blood samples taken from participants with Friedreich's ataxia. Beads (BD Biosciences Trucount™ beads).

Supplementary Methods.

Study Protocol

Title

G-CSF in Friedreich Ataxia study

Full Title

A study of the effects of granulocyte-colony stimulating factor on frataxin expression in a small cohort of patients with Friedreich Ataxia: a proof of concept study.

Version control: GCSF_FRDA_v2
Protocol date: 3.1.18

Trial Identifiers

EudraCT number: **2017-003084-34**

ISRCTN reference:

NHS REC reference: **17/EE/0486**

Sponsor reference: 2823

Funder: Ataxia UK and University of Bristol

Portfolio number: **CPMS 36904**

Table of contents **Page No.**

**1**	**Trial Management**	**5**
1.1	Sponsor	
1.2	Chief Investigator	
1.3	Trial Manager	
1.4	Trial centres	
1.5	Other co-investigators	
1.6	Collaborators	
1.7	Trial statistician	
1.8	Trial Committees	
**2**	**Trial Synopsis**	**7**
**3**	**Trial Flow Diagram**	**7**
**4**	**Timetable and Milestones**	**8**
4.1	Trial activities	
**5**	**Glossary of Terms**	**8**
**6**	**Lay Summary**	**8**
**7**	**Expert Summary**	**9**
**8**	**Introduction**	**9**
8.1	Background	
8.2	Justification for trial design	
**9**	**Trial Objectives and Design**	**10**
9.1	Trial hypothesis	
9.2	Trial design	
9.3	Primary research questions	
9.4	Secondary research questions	
9.5	Population	
9.6	Intervention and placebo	
9.7	Outcomes	
9.8	Expected duration of trial	
9.9	End of trial	
**10**	**Selection and Withdrawal of Subjects**	**12**
10.1	Inclusion criteria	
10.2	Exclusion criteria	
10.3	Selection of participants	
10.4	Selection of sites	
**11**	**Trial Procedures**	**12**
11.1	Baseline assessment	
11.2	Patient follow-up / subsequent assessments	
11.3	Withdrawal of subjects	
**12**	**Trial Medication**	**13**
12.1	Investigational Medicinal Product and comparator	

12.2	Packaging, labelling and dispensing	
12.3	Dosing regimen	
12.4	Drug accountability	
12.5	Subject compliance	
12.6	Concomitant medication	
12.7	Known side effects	
12.8	Return and destruction of medicines	
**13**	**Assessment of Effectiveness**	**14**
**14**	**Assessment of Safety**	**14**
14.1	Definitions	
14.1.1	Adverse Events	
14.1.2	Serious Adverse Events	
14.1.3	Adverse Event Associated With the Use of the Drug	
14.2	Procedure for reporting	
14.2.1	All Adverse Events	
14.2.2	Serious Adverse Events	
14.2.3	Suspected Unexpected Serious Adverse Reaction	
14.3	'Expected' Adverse Events and Reactions	
14.4	Treatment stopping rules	
**15**	**Statistics**	**16**
15.1	Sample size	
15.2	Randomisation	
**16**	**Quality Assurance**	**17**
16.1	Trial monitoring	
16.2	Accuracy of Case Report Forms	
**17**	**Data Handling**	**18**
**18**	**Data Management**	**18**
**19**	**Publication Policy**	**18**
19.1	Definition of authorship	
19.2	Procedure	
19.3	Quality assurance and independent data check prior to publication	
**20**	**Auditing and Inspection**	**19**
20.1	Direct access to source data / documents	
**21**	**Ethics and Regulatory Approvals and Reporting**	**20**
**22**	**Insurance / Indemnity**	**20**
**23**	**Financial Aspects**	**20**
**24**	**Patient and Public Involvement**	**20**
**25**	**Investigative Team Expertise**	**20**
**26**	**Signatures**	**21**

**27**	**Amendment History**	**22**
**28**	**References**	**22**
**29**	**Appendices**	**23**

1. Trial Management

1.1 Sponsor

University of Bristol, Research Governance
Research & Enterprise Development (RED)
University of Bristol
Senate House, level 3
Tyndall Avenue
Bristol BS8 1TH

1.2 Chief Investigator

Dr Alastair Wilkins
Learning and Research Building, Southmead Hospital, Bristol BS10 5NB
P: 0117 4147802
P (Secretary): 0117 4146695
E: alastair.wilkins@bristol.ac.uk

1.3 Trial Manager

Dr Alastair Wilkins

1.4 Trial centres

University of Bristol (single site)

1.5 Other co-investigators

Dr Kevin Kemp
Learning and Research Building, Southmead Hospital, Bristol BS10 5NB
P: 0117 4147802
E: kevin.kemp@bristol.ac.uk

Prof. Yoav Ben-Shlomo
Professor of Clinical Epidemiology
School of Social and Community Medicine
University of Bristol
Canynge Hall, 39 Whatley Road
Bristol BS8 2PS
P: 0117 928 7206
E: y.ben-shlomo@bristol.ac.uk

Dr Chris Metcalfe
Reader in Medical Statistics & Co-director Bristol Randomised Trials Collaboration.
School of Social and Community Medicine
University of Bristol
Canynge Hall, 39 Whatley Road
Bristol BS8 2PS
T: 0117 928 7326
F: 0117 928 7325
E: chris.metcalfe@bristol.ac.uk

Dr James Griffin
Level 4, Bristol Haematology and Oncology Centre,
Horfield Rd, Bristol, BS2 8ED

P:0117 342 1125
E: James.Griffin@uhbristol.nhs.uk

1.6 Collaborators
Nil

1.7 Trial statistician
Prof Ben Shlomo and Dr Metcalfe (as above)

1.8 Trial Committees
As this is a small study investigating the potential of the drug for a wider trial, the Trial Management Group and Trial Steering Committee will comprise the investigators listed above.
In addition, the trial committee will include representation from Ataxia UK, the principle FRDA charity in the UK, and funders of the study.

2. Trial Synopsis

**Trial title**	G-CSF in Friedreich Ataxia study
**Phase**	II
**Sponsor**	University of Bristol, Research Governance<br>Research & Enterprise Development (RED)
**Chief Investigator**	Alastair Wilkins
**ISRCTN**	
**EudraCT No.**	**2017-003084-34**
**REC reference**	
**Medical condition under investigation**	Friedreich Ataxia
**Purpose of trial**	To determine if G-CSF administration to patients with FRDA cause elevation in frataxin gene and protein expression in peripheral blood cells
**Primary objectives**	Determination of frataxin gene and protein expression in peripheral blood cells of FRDA patients who have received G-CSF.
**Secondary objectives**	Monitoring of patients (clinically and blood counts) to determine whether there are any adverse events relating to drug administration.
**Trial design**	Small open label study
**Trial participants**	Participants with Friedreich Ataxia (no controls)
**Outcomes**	Frataxin gene and protein expression
**Sample size**	7
**IMP, dosage, route of administration**	Lenograstim (Granocyte ®) by subcutaneous injection (1.28 million units/kg daily for 5 days)
**Duration of treatment of a subject**	5 day course

3. Trial Flow Diagram

Recuitment of participants via neurology clinics including ataxia clinics

↓

Issuing of information sheets
Discussion of risks involved
Baseline assessment will include clinical examination of pulse, blood pressure and temperature. Baseline bloods will be checked prior to drug administration

→ Consent for study

↓

Treatment commenced (single 5 day course of subcutaneous injection of Lenograstim (Granocyte ®); days 1-5

→ Clinical examination of pulse, blood pressure and temperature on days 1 and 5

↓

Blood samples for analysis of frataxin levels taken at days 5,6,8,10,14 and 19

→ Monitoring bloods taken (full blood count, renal and liver function) on day 19

↓

Final Clinical examination of pulse, blood pressure and temperature on day 19

4. Timetable and Milestones

4.1 Trial activities

Gant Charts

Month	1	2	3	4	5	6	7	8	9
Recruitment	■	■	■						
Drug administration			■	■	■				
Sample analysis						■	■	■	
Data analysis								■	■

5. Glossary of Terms

**BRTC**	Bristol Randomised Trials Collaboration
**CRF**	Case report form
**GCP**	Good clinical practice
**IMP**	Investigational medicinal product
**PIS**	Patient information sheet

6. Lay summary

Friedreich ataxia (FRDA) is an incurable neurological disorder, typically presenting in late childhood, which causes significant disability. There has been much excitement and hope over a number of years that stem cell therapies might provide an effective treatment for a variety of neurodegenerative diseases including FRDA. Indeed, advances in stem cell science over the last decade or more have increased the deliverability of that promise. It is now known that stem cells have multiple functions allowing them to protect against numerous disease processes. Of all the types of stem cell therapies which have been put forward, we believe that bone marrow stem cells hold the most promise, not least as they have been used extensively for many years for other conditions and thus have the best safety profile. There are many potential bone marrow stem cell therapies for FRDA, but utilising the multiple reparative properties of 'a patient's own' stem cells represents the simplest, safest and most readily applicable for an immediate trial. In effect, such a therapy represents a form of cellular mobilisation- making stem cells travel to sites of injury in the body to repair the damage- which has been used in other conditions with effect, not least our own group's trials of autologous bone marrow transplantation for multiple sclerosis. An alternative to transplanting bone marrow cells, without the need for bone marrow harvest and infusions, would be to use bone marrow stem cell mobilising drugs, such as GCSF (granulocyte colony stimulating factor) which is in common usage in haematological conditions, as well as in healthy people prior to bone marrow donation. This is a drug that activates stem cells within the bone marrow and induces them to circulate around the body. We believe that increasing the circulation of the body's own stem cells is a promising approach for therapeutic success and we hypothesise that their potential mechanisms of actions are multiple.

Experimental studies undertaken within our laboratories clearly indicate bone marrow stem cells protect nerve cells and induce repair of the nervous system. We have recently completed a major study of a mouse model of FRDA in which bone marrow stem cell mobilising drugs protected mice from neurological damage. These drugs have huge potential in neurodegenerative conditions and represent a novel therapeutic possibility for FRDA.

The next stage in our research is to further understand whether GCSF induces similar changes in humans with FRDA. Since the major studies we have performed have been in mice, we want to make sure the response is the same in human cells. This study will also help us to define the dosage and selection of patients for our forthcoming clinical trial in FRDA. This protocol will study a small number of patients with the condition and will involve administration of GCSF (at identical doses to those given to 'healthy' people prior to bone bone marrow donation) for a short period of time. We will define whether administration of the drug leads to changes in blood markers which would indicate a positive response to the drug. The study will also allow us to decide what blood markers we can monitor in the subsequent trial. This has not been studied before and is a vital step in the development of a stem cell research trial. Once information has been obtained from this study, a larger trial of GCSF in FRDA can be developed.

7. Expert Summary

Despite our increased understanding of how the genetic abnormality in Friedreich ataxia (FRDA) causes disease, there are still no treatments available to reduce long term disability. We have been studying stem cell-mediated neuroprotection in FRDA models for a number of years and have recently completed an exciting study of cytokine-mediated stem cell-mobilisation therapy in an animal FRDA model. We have shown that a bone marrow stem cell-mobilising cytokine called granulocyte colony stimulating factor (GCSF) is very effective in reversing symptoms, pathological changes and biochemical abnormalities in the model. We have therefore begun to plan for a phase 2 trial of GCSF in FRDA. The safety profile and pharmacokinetics of GCSF are well established, being used extensively in 'healthy' bone marrow donors. However, prior to starting a trial we wish to establish the effect of this agent in people with FRDA in order to define whether GCSF induces similar changes in biochemical profiles which occur in the models. We will conduct initial studies of the effect of GCSF administration in a small number of patients with FRDA. This will allow for refinement of trial protocol and improved patient selection, as well as defining biomarkers for treatment response, with the ultimate aim of developing a novel FRDA therapy.

8. Introduction

8.1 Background

For a number of years, we have been studying potential new therapies for Friedreich Ataxia (FRDA)[1-8]. FRDA is one of the commonest inherited neurological disorders and is caused by a genetic abnormality in the frataxin gene leading to reduced levels of this key mitochondrial protein. Symptoms usually start in childhood and lead to progressive disability and premature death. Currently there are no effective treatments to prevent or slow disease progression. We have completed a major MRC-funded study of bone marrow stem cell (BMSC) mobilisation in a mouse model of FRDA[6]. Importantly, as part of this study we showed that treatment with the stem cell mobilising agent- granulocyte colony stimulating factor (GCSF)- led to significant functional, pathological and biochemical improvements in the mice, associated with increased frataxin levels in the nervous system of frataxin-deficient mice.

Disease modification in FRDA and associated degenerative ataxic conditions represents a major unmet clinical need. Having completed the animal model studies we have established the rationale for a novel human trial of disease modification using GCSF in FRDA.

There are currently no licenced disease modifying therapies available for patients with FRDA. Several speculative strategies to reduce disease progression in FRDA have been proposed and are in clinical trial. These include therapies aimed at increasing levels of the protein frataxin (which is deficient in FRDA) and agents which may reduce the consequences of frataxin deficiency (e.g. iron overload). No phase II or III trial has so far shown reductions in neurological disability. Preclinical experimental models suggest the mechanism of action of the majority of these drugs is to prevent further tissue injury (i.e. neuro-protective). None of the current trials in development for FRDA suggest a potential neuro-reparative mechanism which is the main competitive advantage of our proposed trial.

We have formed a trial steering group and have been aided by Research Enterprise and Development at the University of Bristol. The trial steering group includes representatives from Bristol Randomised Trials Collaboration and we are in the process of designing a phase 2 clinical trial.

Our studies show that GCSF can reverse pathology in the FRDA model. Elevation of frataxin in the central and peripheral nervous system occurs which is a key strategy in FRDA therapeutics. However, GCSF treatment also caused widespread integration of bone marrow cells and recruitment of endogenous stem cell populations which contribute to restoration of normal tissue architecture in diseased areas of the nervous system. Importantly, GCSF therapy given after onset of clinical disease leads to improvement in clinical scores and reversal of neurological deficits. Thus the treatment may have not only neuroprotective effects, but also neuro-reparative effects giving it a significant competitive advantage over other agents in trial. Importantly, the effects seen in the model of FRDA may also be generalizable to the treatment of other degenerative ataxic conditions. In addition, GCSF is licenced for use in haematological conditions, allowing rapid translation to clinical trialling for FRDA[9-12].
Clearly, however, efficacy having thus far only been demonstrated in rodent models, key experiments providing evidence of comparable GCSF effects in human subjects is required prior to application for trial funding; these vital studies would increase the likelihood of trial grant support and, importantly, aid trial design. We, therefore, wish to perform a small study of administration of GCSF to patients with FRDA to determine changes in peripheral blood markers linked to disease.

8.2 Justification for trial design

This is a small study of 7 patients with FRDA which is designed to determine if biochemical changes induced by GCSF, and noted in mouse model studies, are replicated in humans with the condition. At the same time, initial safety data will be obtained concerning the administration of GCSF in people with FRDA. There is extensive human safety data on the use of GCSF in 'healthy' people. Although there are no theoretical concerns about GCSF use in FRDA, the study will provide some useful preliminary safety data.
We consider this study to be an important pre-requisite to a larger phase 2 trial of GCSF in FRDA.

9. Trial Objectives and Design

9.1 Trial Hypothesis

GCSF administration to patients with FRDA causes elevation in frataxin gene and protein expression in peripheral blood cells.

9.2 Trial Design

This will be a small study. 7 patients with FRDA will be given a single 5 day course of lenograstim (Granocyte ®) by subcutaneous injection (1.28 million units/kg daily for 5 days; which is the standard course for 'healthy volunteers prior to bone marrow harvest for bone marrow donation).

- Participants will be selected from regional ataxia clinical services in Bristol (the chief investigator runs specialist ataxia services in the region). Participants will be selected on the basis of an established genetic diagnosis of FRDA. If possible patients with a range of trinucleotide repeat expansion levels will be selected. In the event that not enough patients can be recruited through this route, a request that people with the condition are referred to the chief investigator's clinic. This request will be coordinated through Ataxia UK, the charity funding the research and which is part of the trial steering committee.
- Full consent will be taken and discussion of potential risks (by chief investigator)
- Baseline blood tests for full blood count, liver and renal function will be performed within one month of starting the drug treatment. Routine blood monitoring tests are performed as part of standard care of patients with FRDA.
- Baseline observations (pulse, blood pressure and temperature) will be performed prior to first injection. Patient's weight in kilograms will be determined prior to the first investigation in order to determine the dose of lenograstim to be administered. Results of baseline blood tests will be checked.
- Patients will be given a single 5 day course of lenograstim (Granocyte ®) by subcutaneous injection (1.28 million units/kg daily for 5 days). Patients will have daily injections administered by medical/ nursing staff. This will occur in the Bristol Brain Centre which is set up specifically to carry out clinical

neurological trials. Nursing support from the Bristol Haematology and Oncology Centre to train neurology nurses and patients administration of the drug has been agree by the haematologist on the trial committee.

- Blood will be taken on day 5, 6, 8, 10, 14 and 19 (from the start of the drug dosing, so blood sampling will occur initially on the last day of dosing) for determination of frataxin levels.
- At time of administration, assessment for any adverse events from GCSF administration will be made.
- Baseline observations (pulse, blood pressure and temperature) and routine laboratory blood work-up (full blood count, liver and renal function) will be taken 2 weeks after drug administration has finished (day 19).
- Frataxin levels in peripheral blood mononuclear cells will be determined by RT-PCR (as above) and also by using commercially available frataxin immunoreactivity assays (used in previous FRDA studies and trials) in order to determine the validity of this approach in the future trial. This will provide important pharmacodynamic data relating to GCSF dosage. Peripheral blood mononuclear cells are known to express G-CSF receptors[13].
- In addition, samples will be used to measure frataxin protein expression by immunoblotting and ELISA. Other candidate molecules related to frataxin's function may also be analysed from the samples that are taken.

The standard care of patients with FRDA in neurology clinics typically includes annual follow-up appointment with discussion of neurological problems and neurological examination. Patients will typically have routine annual blood tests (for liver, kidney function and full blood count) and will have echocardiography every 2 years (to assess for cardiomyopathy).
Therefore the additional burden for patients involved in the study will be attendance at hospital on 10 days over the course of a 19-day period. The standard of care will not otherwise be affected and patients will continue to receive standard care in neurology clinics for routine aspects of their neurological care.
The safety of Granocyte administration to people with FRDA is of paramount importance. Patients will be assessed clinically during and 2 weeks after drug administration has finished. The trial steering committee includes a haematologist who advises on the safety of G-CSF administration. There is a wealth of safety data from the British Bone Marrow registry (amongst others) which suggests that G-CSF administration in non-haematological patients (i.e. healthy bone marrow donors) is safe[10,11]. We have access safety to data from the British Bone Marrow registry.

9.3 Primary Research Questions
Does GCSF administration to patients with FRDA cause elevation in frataxin gene and protein expression in peripheral blood cells?

9.4 Secondary Research Questions
Is GCSF administration (single course) safe in FRDA patients?

9.5 Population
Patients with genetically confirmed diagnosis of FRDA.

9.6 Intervention and placebo
Single 5 day course of lenograstim (Granocyte ®) by subcutaneous injection (1.28 million units/kg daily for 5 days). There will be no placebo.

9.7 Outcomes

Primary outcomes:
Frataxin gene and protein expression in peripheral blood cells of FRDA patients who have received granocyte.

Secondary outcomes:
Safe administration of the drug, monitored by clinical monitoring and laboratory blood monitoring.

Source data will include: documents of clinical assessment of patient (pulse, blood pressure and temperature); reports of full blood count, liver and renal function; and documentation of blood taking for trial samples. Source data will be documented in the electronic case report form (eCRF).

9.8 Expected Duration of Trial
7 patients will be treated and monitored for a period of 19 days each, over a course of approximately 4 months.

9.9 End of Trial
The trial will end after recruitment, drug administration, sample collection and post-drug administration monitoring period has been completed.

<u>10. Selection of Subjects</u>

10.1 Inclusion Criteria (all must apply)
Genetic diagnosis of FRDA
Age of over 18

10.2 Exclusion Criteria (presence of any warrants exclusion)

Any contraindication to GCSF therapy:

- known hypersensitivity to lenograstim or to any of the excipients;
- patients receiving concurrent cytotoxic chemotherapy;
- patients with myeloid malignancy, *de novo* acute myeloid leukaemia aged below 55 years, and/or *de novo* acute myeloid leukaemia with good cytogenetics, i.e. t(8 ;21), t(15 ;17) and inv (16).
- severe congenital neurtropaenia (Kostmann's syndrome) with abnormal cytogenetics

Pregnancy, breastfeeding or lactation
Significant abnormalities on baseline bloods (full blood count, renal and liver function)
Previous diagnosis of haematological disorder (including malignancy)
Previous history of splenomegaly
Previous history of autoimmune disease
Previous history of pulmonary infiltrates, pulmonary fibrosis or haemoptysis

10.3 Selection of Participants
Patients attending neurology clinics (including ataxia clinics) in North Bristol NHS trust.

10.4 Selection of Sites
Single site

<u>11. Trial Procedures</u>

11.1 Baseline assessment
Baseline assessment will include clinical examination of pulse, blood pressure and temperature. Baseline bloods will be performed (full blood count, renal and liver function) prior to drug administration.

11.2 Patient follow-up / subsequent assessments
Patients will be assessed:

Clinically: by discussing whether there have been any side effects, measuring pulse, temperature and blood pressure
Blood work-up: full blood count, renal and liver function.
Bloods for trial analysis

At the following points:

Day	1	2	3	4	5	6	8	10	14	19
**Drug adminis-tration**	✓	✓	✓	✓	✓					
**Clinical assess-ment**	✓				✓					✓
**Blood work-up**	✓					✓				✓
**Blood for trial analysis**					✓	✓	✓	✓	✓	✓

11.3 Withdrawal of Subjects
Subjects will be able to withdraw at any point if he or she requests. In addition, any significant abnormality on clinical assessment or blood monitoring will be a cause for withdrawal. If samples have been obtained from subjects who subsequently withdraw from the trial, these samples will have been anonymised and therefore will not be withdrawn from the research, but no further samples or data will be collected from that subject.

12. Trial Medication

12.1 Investigational Medicinal Product and comparator
Lenograstim (Granocyte ®) manufactured by Chugai Pharma UK.

12.2 Packaging, labelling and dispensing
As the study is not blinded or randomised, the drug will be packaged, labelled and dispensed in its usual form.

12.3 Dosing Regimen
Lenograstim (Granocyte ®) by subcutaneous injection (1.28 million units/kg daily for 5 days)

12.4 Drug accountability
The University of Bristol (UoB) has a Service Level Agreement in place with a local NHS Trust (UH Bristol). As part of this, UH Bristol will provide guidance and SOPs.

12.5 Subject compliance
Drug will be administered by registered nurse or doctor during the course of the trial.

12.6 Concomitant Medication
Any medication taken by the participant will be disclosed and decision made as to whether there may be any drug interaction with Lenograstim (Granocyte ®). If such an interaction is thought likely, the participant will not be given the drug and be withdrawn from the study.

12.7 Known side effects

The most frequently reported undesirable effects are transient and mild to moderate: pain, bone pain, back pain, asthenia, fever, headache and nausea and increased liver function tests (which typically resolve on cessation of therapy). Injection site reactions can occur.
Rare pulmonary adverse reactions have been reported such as dyspnoea, hypoxia or haemoptysis, including very rarely Acute Respiratory Distress Syndrome (ARDS)
There is a small increase in the risk of autoimmune disease (notably rheumatological conditions) but this seems to be in those with established family history of those diseases. The previously cited risk of splenomegaly and splenic rupture seems not to be proven and patients are no longer warned of this risk (British Bone Marrow registry guidelines). Capillary leak syndrome has been reported rarely (≥ 1/1000 to < 1/100; mostly in cancer patients undergoing chemotherapy) after G-CSF administration, and is characterised by hypotension, hypoalbuminaemia, oedema and hemoconcentration. Skin disorders (cutaneous vasculitis; erythema nodosum; pyoderma gangrenosum; Lyell's syndrome; Sweet's syndrome) have been reported very rarely (<1/10000).

12.8 Return and destruction of medicines
Any excess medicines will be destroyed in clinical repositories.

13. Assessment of Effectiveness

Primary outcomes:
Frataxin gene and protein expression in peripheral blood cells of FRDA patients who have received granocyte will be measured to determine drug response.

Secondary outcomes:
Monitoring of patients (clinically and blood counts) to determine whether here are any adverse events relating to drug administration.

Source data will include: documents of clinical assessment of patient (pulse, blood pressure and temperature); reports of full blood count, liver and renal function; and documentation of blood taking for trial samples. Source data will be documented in the electronic case report form (eCRF).

14. Assessment of Safety

14.1 Definitions

14.1.1 Adverse Events (AE)
AEs are defined as any untoward medical occurrence in a clinical study participant. An AE does not necessarily have to have a causal relationship with the study treatment. An AE can therefore be any unfavourable and unintended sign (including an abnormal finding), symptom or disease temporally associated with the use of a medicinal (investigational) product, whether or not related to the medicinal (investigational) product. (International Conference on Harmonisation [ICH] definition). This includes any occurrence that is new in onset or aggravated in severity or frequency from the baseline condition, or abnormal results of diagnostic procedures, including laboratory test abnormalities.
All AEs will be recorded in the Case Report Form (CRF) for the duration of the participant's involvement in the study.

14.1.2 Serious Adverse Events (SAEs)
A SAE is defined by ICH as any untoward medical occurrence that at any dose of the study medication meets any of the following conditions:

1. **Results in the death of the participant**
2. **Is life-threatening**
   The term "life-threatening" refers to an event in which the participant was at risk of death at the time of the event; it does not refer to an event which hypothetically might have caused death if it were more severe.

3. **Requires inpatient hospitalisation or prolongation of existing hospitalisation**
   For any event that may not be immediately life-threatening or result in death or hospitalisation, but may jeopardise the participant or may require intervention to prevent one of these outcomes (*insert a study specific example*), the CI should exercise his/her scientific and medical judgement to decide whether or not such an event requires expedited reporting to UH Bristol (who acts on behalf of the Sponsor in these instances).
4. **Results in persistent or significant disability / incapacity**
   Any event that seriously disrupts the ability of the participant to lead a normal life, in other words leads to a persistent or permanent significant change, deterioration, injury or perturbation of the participant's body functions or structure, physical activity and/or quality of life.
5. **Is a congenital anomaly / birth defect**
   Exposure to the study drug before conception (in men or women) or during pregnancy that resulted in an adverse outcome in the child.
6. **Other medical events**
   Medical events that may jeopardise the subject or may require an intervention to prevent a characteristic or consequence of a SAE. Such events are referred to as 'important medical events' and are also considered as 'serious' in accordance with the definition of a SAE.

14.1.3 Adverse Event Associated With the Use of the Drug
An AE is considered to be associated with the use of the drug if the attribution is possible, probable, or very likely by the definitions listed below.

- **Not related:** An AE that is not related to the use of the drug.
- ***Doubtful:*** An AE for which an alternative explanation is more likely, e.g., concomitant drug(s), concomitant disease(s), or the relationship in time suggests that a causal relationship is unlikely.
- ***Possible****:* An AE that might be due to the use of the drug and for which an alternative explanation, e.g., concomitant drug(s), concomitant disease(s), is inconclusive. The relationship in time is reasonable and therefore, the causal relationship cannot be excluded.
- ***Probable****:* An AE that might be due to the use of the drug. The relationship in time is suggestive (e.g., confirmed by IMP withdrawal). An alternative explanation is less likely, e.g., concomitant drug(s), concomitant disease(s).
- ***Very likely:*** An AE that is listed as a possible adverse reaction and cannot be reasonably explained by an alternative explanation, e.g., concomitant drug(s), concomitant disease(s). The relationship in time is very suggestive (e.g., it is confirmed by IMP withdrawal and re-introduction).

14.2.2 Procedures for reporting

<u>14.2.1 All Adverse Events</u>

All AEs will be reported by the Chief Investigator from the time a signed and dated informed consent form is obtained until completion of the last study-related procedure. Those occurrences meeting the definition of SAEs must be reported using the Serious Adverse Event Form (*available from UH Bristol)*, including SAEs spontaneously reported to the Investigator within 30 days after the participant has completed the study (including post study follow-up). UH Bristol, on behalf of the Sponsor, will evaluate any safety information that is spontaneously reported by a CI beyond the time frame specified in the protocol.
All AEs, regardless of seriousness, severity, or presumed relationship to study drug, must be recorded in the source document and the CRF, together with any measures taken. CIs must record in the CRF their opinion concerning the relationship of the adverse event to study therapy. UH Bristol, on behalf of the Sponsor, assumes responsibility for appropriate reporting of adverse events to the regulatory authorities.

<u>14.2.2 Serious Adverse Events (SAEs)</u>

All SAEs must be reported to the UH Bristol contact (fax 0117 3420239 or research@uhbristol.nhs.uk) by investigational staff within 24 hours of their knowledge of the event.
All SAEs that have not resolved by the end of the study, or that have not resolved upon discontinuation of the participant's participation in the study, must be followed until any of the following occurs:

- the event resolves

- the event stabilizes
- the event returns to baseline, if a baseline value is available
- the event can be attributed to agents other than the study drug or to factors unrelated to study conduct
- when it becomes unlikely that any additional information can be obtained (participant or health care practitioner refusal to provide additional information, lost to follow-up after demonstration of due diligence with follow-up efforts)

The death of a participant is considered an SAE, as is any event requiring hospitalization (or prolongation of hospitalization) that occurs during the course of a participant's participation. Exceptions to this are hospitalizations for:

- social reasons in absence of an adverse event
- the in-clinic protocol procedures
- surgery or procedure planned before entry into the study (must be documented in the CRF)

<u>14.2.3 Suspected Unexpected Serious Adverse Reaction (SUSAR)</u>

All relevant information about a SUSAR which occurs during the course of the study and is fatal or life-threatening will be reported within 7 days to the MHRA and the relevant ethics committee by UH Bristol, on behalf of the Sponsor. The expectedness of an adverse event will be determined by whether or not it is listed in the Summary of Product Characteristics.

All relevant information about a non-fatal or life-threatening SUSAR which occurs during the course of the study will be reported within 15 days to the MHRA and the relevant ethics committee by UH Bristol, on behalf of the Sponsor. The expectedness of an adverse event will be determined by whether or not it is listed in the Summary of Product Characteristics.

14.3 'Expected' Adverse Events and Reactions

Side effects	% Adverse Events in IMP arm	% AEs in placebo arm	Difference
bone pain/ hip girdle pain	approximately 10%	-	-
Elevated liver function tests (LDH, ALT, AST, ALP; which typically resolve on cessation of therapy)	approximately 10%	-	-
Headache, asthenia	approximately 10%	-	-
Leucocytosis and thrombocytopaenia (which typically resolve on cessation of therapy)	approximately 10%	-	-
Injection site reaction	approximately 1-10%		
Abdominal pain	approximately 1-10%		
autoimmune disease (notably rheumatological conditions)	<1%		
Capillary leak syndrome (typically patients receiving concomitant chemotherapy)	Approximately 0.1%		
Pulmonary infiltrate/ fibrosis/ oedema; interstitial pneumonitis	<0.01%		
Skin disorders (cutaneous vasculitis; erythema nodosum; pyoderma gangrenosum; Lyell's syndrome; Sweet's syndrome)	<0.01%		

Side effects	% Adverse Events in IMP arm	% AEs in placebo arm	Difference
Anaphylaxis	<0.01%		

14.4 Treatment Stopping Rules
Any SAE reported during the course of treatment will lead to treatment being stopped and the participant being withdrawn from the study. Monitoring processes will continue at the same time points as described above in such an instance.

15. Statistics

15.1 Sample size
This is a small 'proof of principle study of 7 patients prior to larger phase 2/3 studies.

15.2 Data Analysis
Frataxin levels in peripheral blood mononuclear cells will be determined by RT-PCR (as above) and also by using commercially available frataxin immunoreactivity assays (used in previous FRDA studies and trials) in order to determine the validity of this approach in the future trial. This will provide important pharmacodynamic data relating to GCSF dosage.

16. Quality Assurance
A risk based monitoring plan will be prepared in collaboration with University of Bristol Research Enterprise and Development and University Hospitals, Bristol. A quality assurance document to reflect steps to be taken throughout the study will be prepared.

16.1 Trial Monitoring
Study monitoring will be undertaken on behalf of the Sponsor by UH Bristol using their monitoring standard operating procedure:
http://www.uhbristol.nhs.uk/research-innovation/information-for-researchers/setting-up-and-running-a-clinical-research-study/what-to-do-when-approval-is-received/

16.1.2 Before the Study
The CI will work with UH Bristol to develop risk based monitoring plan. Non-regulatory documents, such as SOPs, are reviewed by UH Bristol in preparation for study start up to ensure that key protocol requirements are captured not just in the regulatory documentation but also in the working documents that the research team is planning to use.

The CI will allow the monitor to visit the site and facilities where the study will take place in order to ensure compliance with the protocol requirements.

16.1.3 During the Study
The CI will allow the monitor and/or the Sponsor to:
- Inspect the site, the facilities and the material used for the study
- Meet all members of his/her team involved in the study
- Consult all of the documents relevant to the study
- Check that the CRFs have been filled out correctly
- Directly access source documents for comparison of data therein with the data in the CRFs
- Verify that the study is carried out in compliance with the protocol and local regulatory requirements
- Carry out study monitoring at regular intervals, depending on the recruitment rate, and arranged between the CI and monitor
- All information dealt with during these visits will be treated as strictly confidential

- Respond to monitoring report

16.1.4 Access to Source Data / Documents

- The CI will allow monitors (from UH Bristol on behalf of the Sponsor), persons responsible for monitoring, representatives of the Ethics Committee and of the Regulatory Authorities to have direct access to source data/documents. This is reflected in the Participant Information Sheet (PIS).

16.2 Accuracy of Case Report Forms

The study Case Report Form (CRF) is the primary data collection instrument for the study. All data requested on the CRF will be recorded and checked. All missing data will be explained. If a space on the CRF is left blank because the procedure was not done or the question was not asked, "N/D" will be inserted. If the item is not applicable to the individual case, "N/A" will be inserted. All entries will be printed legibly in black ink. If any entry errors are made, to correct such an error, a single straight line will be drawn through the incorrect entry and the correct data entered above it. All such changes will be initialled and dated. Pencil and correction fluid will not be used anywhere on the CRF. If it is not clear why the change has been made, an explanation will be written next to the change.

Data collected on each subject will be recorded by the Principal Investigator, or his designee (as noted on the Site Responsibilities Sheet). The Principal Investigator will be responsible for the timing, completeness, legibility and accuracy of the CRF and he/she will retain a copy of each completed form. The Principal Investigator will allow study staff access to any required background data from such records (source data e.g. medical records) on request.

Each patient enrolled into the study must have the correct CRFs completed and signed by the Principal Investigator (or designee). This also applies to those patients who failed to complete the study. All data submitted on CRFs must be verifiable in the source documentation or the discrepancies explained.

If a patient withdraws from the study during the treatment phase, the reason must be noted on the Treatment Completion/Withdrawal Form and the patient must be followed-up as per protocol. If the patient withdraws their consent to any further participation in the study (treatment and follow-up) this must be recorded and no follow-up is required.

17. Data Handling

Data will be entered on to a Microsoft Access spreadsheet using a double data entry system, enabling generation of a single dataset following a process of data comparison. Further electronic data capture methods may be explored once the study is under way.

18. Data Management

Data will be collected and stored in accordance with the Data Protection Act 1998. Names, addresses and other personal identifiers will be stored (in both paper and electronic formats) separately from questionnaire and clinical data to prevent identification of research records. The anonymised research records (paper) will be stored in locked filing cabinets within a locked office. Electronic records will be stored on a restricted access database on a secure University of Bristol server backed up to an external hard drive with 256-bit Secure Socket Layer (SSL) encryption which will remain in the School of Clinical Sciences, University of Bristol.

19. Publication Policy

19.1 Definition of authorship

An author is considered to be someone who has made substantive *intellectual* contribution to a study. Many journals consider it best practice that everyone who is listed as an author should have made a substantial, direct, intellectual contribution to the work. Honorary or guest authorship is not acceptable.

19.2 Procedure

The baseline criteria for this research for both authorship and acknowledgments for peer reviewed publications and conference contributions is that:

1. Authors must meet *all* of the following criteria:
    i. substantial contributions to conception and design, acquisition of data, or analysis and interpretation of data
    ii. drafting the article or revising it critically for important intellectual content
    iii. final approval of the version to be published
2. No-one should be omitted from the authorship list if he/she meets the three criteria in 1 above.
3. Some journals allow authorship of multi-centre projects to be attributed to a group. However all members of the group who are named as authors must still fully meet the above criteria for authorship in 1 above.
4. Other collaborators or members of the research group who may have contributed to some but not all of the criteria in 1 above will be listed in the Acknowledgments (see 6 below).
5. The individual authors will jointly make decisions about authorship before submitting the manuscript for publication. The lead author, corresponding author or the guarantor must be prepared to explain the presence and order of these individuals to the editor of a journal. Authorship and order of authorship (see 7 below) will be agreed in advance, in the early stages of the research.
6. All contributors who do not meet the criteria for authorship will be listed in an Acknowledgments section. Examples of those who might be acknowledged include:
    - persons who have contributed materially to the paper but whose contributions do not justify authorship. These may be listed under such headings as "participating investigators" and their function or contribution should be described - for example, "served as scientific advisors," "critically reviewed the study proposal," or "collected data/material". Because readers may infer their endorsement of the data and conclusions, these persons must give written permission to be acknowledged
    - a person who provided purely technical help, provided general comments on the manuscript or writing assistance, or a departmental chair who provided general support
    - editors can ask corresponding authors to declare whether they had assistance with study design, data collection, data analysis, or manuscript preparation. Authors should therefore disclose in the Acknowledgements section the identity of any individuals who provided this assistance and any entities that supported the work in the published article
    - financial support should also be acknowledged and, if appropriate, the grant identified
    - material or logistical support, in particular giving recognition to support provided in developing countries, should always be acknowledged
7. Order of authorship
    - the authors shall decide the order of authorship together. Contributors should discuss authorship issues frankly at the start of the work for each anticipated publication and not wait to raise concerns at submission time
    - authors shall specify in their manuscript a description of the contributions of each author and how they have assigned the order in which they are listed so that readers can interpret their roles correctly
    - the corresponding author or guarantor shall prepare a concise, written description of how the order of authorship was decided
    - examples of authorship order include:
        - descending order of contribution
        - placing the person who took the lead in writing the manuscript or doing the research first and the most experienced contributor in the field last
        - alphabetical
        - random order
8. If an individual leaves the project the question of contribution to publications and authorship should be discussed in advance of their departure to minimise misunderstandings and to agree how this will be managed.

<u>19.3 Quality assurance and independent data check prior to publication</u>

Responsibility for data management in publications will be performed by Dr Metcalfe (co-investigator).
In addition an independent proof reading process during the final stages of the publication acceptance process will be developed to ensure data accuracy of the publication.

20. Auditing and Inspection

20.1 Direct Access to Source Data / Documents
All source documents and other essential study records will be stored for a period of 15 years after the end of the trial. No study-related records should be destroyed unless or until the sponsor gives authorisation to do so. Any medical case notes containing trial-related information should be identified by a label "Keep until dd/mm/yyyy" where the date given is 15 years after the last participant's final study visit. Following completion of analysis, the sponsor will be responsible for archiving the study data at a secure location.

21. Ethics and Regulatory Approvals and Reporting

Before the start of the trial, approval will be sought from a REC for the trial protocol, informed consent forms and other relevant documents e.g. advertisements and GP information letters

Substantial amendments that require review by REC will not be implemented until the REC grants a favourable opinion for the trial (note that amendments may also need to be reviewed and accepted by the MHRA and/or NHS R&D departments before they can be implemented in practice at sites)

All correspondence with the REC will be retained in the Trial Master File/Investigator Site File

An annual progress report (APR) will be submitted to the REC within 30 days of the anniversary date on which the favourable opinion was given, and annually until the trial is declared ended

The Chief Investigator will notify the REC of the end of the trial

If the trial is ended prematurely, the Chief Investigator will notify the REC, including the reasons for the premature termination

Within one year after the end of the trial, the Chief Investigator will submit a final report with the results, including any publications/abstracts, to the REC

22. Insurance / Indemnity

This study will be sponsored by the University of Bristol. In the event that a participant is harmed during the trial, the University has arranged Clinical Trials Liability Insurance. This provides "No Fault" (non-negligent harm) compensation to research participants when on the balance of probabilities bodily injury, death, disease, illness or disability (including exacerbation of an existing condition) is caused by the administration to or use by the research participant of the drug involved in the trial or is directly due to participation in the Clinical Trial.

23. Financial Aspects

Funding has been obtained from Ataxia UK, the main UK charity for FRDA.

24. Patient and Public Involvement

We are in the process of convening a Patient and Public Involvement (PPI) group for the larger trial. The PI is a neurologist who treats patients with ataxia and often discusses needs and research directions with people with ataxia and carers. At the forefront f discussion is the need to provide a therapy which can reduce the burden of disability in the long term and this has driven pre-clinical studies and the current trial development. The PI has discussed these aspects with the main research charity for ataxia in the UK (Ataxia UK, who are funding the current study) and has presented at Ataxia UK patient and carer information days on a number of occasions.

25. Investigative team expertise

The Principal investigator is a consultant neurologist who has experience (as local investigator and co-investigator) of clinical trials (commercial and non-commercial). He has expertise in ataxia and sees regional referrals for ataxia (including FRDA). He is part of the MS and Stem cell laboratories, University of Bristol which has built up extensive experience of pre-clinical and clinical research. In the unit, there are a number of trials running relating to stem cell therapies, as well as commercial trials. In addition, the laboratories have developed extensive experience of laboratory investigations in the field of degenerative ataxias, including studies of Friedreich Ataxia. We have published extensively in the field of neurodegenerative conditions, particularly Friedreich ataxia and multiple sclerosis.

Dr Kemp has over 10 years' experience studying stem cells, ataxia models and molecular biology techniques. This project builds on previous studies funded by MRC and Ataxia UK studying the properties of stem cells in relation to Friedreich ataxia and links in to our recently published animal model studies.

Professor Ben-Shlomo and Dr Metcalfe are part of the Bristol Randomised Trials Collaboration and have extensive experience in running and interpreting clinical trials.

Dr Griffin is a consultant haematologist who has a joint appointment between NHS Blood and Transfusion and Bristol Haematology and Oncology Centre (BHOC). He is designated Individual for the Human Tissue Authority for BHOC and is closely involved in the Bone Marrow donor scheme.

26. Signatures

_______________________________ _____________

Chief Investigator Date

Print name

_______________________________ _____________

Sponsor Date

Print name

27. Amendment History

Amendment No.	Protocol Version No.	Details of the changes made	Date Issued

28. References

1 Dey, R. *et al.* Human mesenchymal stem cells increase anti-oxidant defences in cells derived from patients with Friedreich's ataxia. *Cerebellum* **11**, 861-871, doi:10.1007/s12311-012-0406-2 (2012).

2 Kemp, K., Dey, R., Cook, A., Scolding, N. & Wilkins, A. Mesenchymal Stem Cell-Derived Factors Restore Function to Human Frataxin-Deficient Cells. *Cerebellum*, doi:10.1007/s12311-017-0860-y (2017).

3 Kemp, K. *et al.* Fusion between human mesenchymal stem cells and rodent cerebellar Purkinje cells. *Neuropathol Appl Neurobiol* **37**, 166-178, doi:10.1111/j.1365-2990.2010.01122.x (2011).

4 Kemp, K. *et al.* Mesenchymal stem cell-secreted superoxide dismutase promotes cerebellar neuronal survival. *J Neurochem* **114**, 1569-1580, doi:10.1111/j.1471-4159.2009.06553.x (2010).

5 Kemp, K. *et al.* Mesenchymal stem cells restore frataxin expression and increase hydrogen peroxide scavenging enzymes in Friedreich ataxia fibroblasts. *PLoS One* **6**, e26098, doi:10.1371/journal.pone.0026098 (2011).

6 Kemp, K. C. *et al.* Cytokine therapy-mediated neuroprotection in a Friedreich's ataxia mouse model. *Ann Neurol* **81**, 212-226, doi:10.1002/ana.24846 (2017).

7 Kemp, K. C. *et al.* Purkinje cell injury, structural plasticity and fusion in patients with Friedreich's ataxia. *Acta Neuropathol Commun* **4**, 53, doi:10.1186/s40478-016-0326-3 (2016).

8 Wilkins, A. *et al.* Human bone marrow-derived mesenchymal stem cells secrete brain-derived neurotrophic factor which promotes neuronal survival in vitro. *Stem Cell Res* **3**, 63-70, doi:10.1016/j.scr.2009.02.006 (2009).

9 Tarella, C. *et al.* Consistent bone marrow-derived cell mobilization following repeated short courses of granulocyte-colony-stimulating factor in patients with amyotrophic lateral sclerosis: results from a multicenter prospective trial. *Cytotherapy* **12**, 50-59, doi:10.3109/14653240903300682 (2010).

10 Martino, M. *et al.* Long-term safety of granulocyte colony-stimulating factor in normal donors: is it all clear? *Expert Opin Biol Ther* **12**, 609-621, doi:10.1517/14712598.2012.674937 (2012).

11 Shaw, B. E., Confer, D. L., Hwang, W. & Pulsipher, M. A. A review of the genetic and long-term effects of G-CSF injections in healthy donors: a reassuring lack of evidence for the development of haematological malignancies. *Bone Marrow Transplant* **50**, 334-340, doi:10.1038/bmt.2014.278 (2015).

12 Heussner, P. *et al.* G-CSF in the long-term treatment of cyclic neutropenia and chronic idiopathic neutropenia in adult patients. *Int J Hematol* **62**, 225-234 (1995).

13 Aritomi, M. *et al.* Atomic structure of the GCSF-receptor complex showing a new cytokine-receptor recognition scheme. *Nature* **401**, 713-717, doi:10.1038/44394 (1999).

29. Appendices

Appendix I Definitions of severity and causality of adverse events

The local principal investigator must assess the severity of an adverse event according to this scale:

- Mild: An event that is easily tolerated by the patient, causing minimal discomfort and not interfering with everyday activities.
- Moderate: An event that is sufficiently discomforting to interfere with normal everyday activities.
- Severe: An event that prevents normal everyday activities.

The investigator should also assess the causality of the adverse event, paying particular consideration to the pharmacodynamics and pharmacokinetics of the treatment, the time course of the events in relation to the treatment and the possible presence of another cause.

The relationship of the adverse event to treatment should be graded as:

- Not related: the relationship of the onset of the event relative to the administration of the trial medication is not reasonable or there is another cause which can explain the occurrence of the event.
- Unlikely: the relationship of the onset of the event relative to the administration of the trial medication is likely to have another cause which can explain the occurrence of the event.
- Possibly related: the relationship of the onset of the event relative to the administration of the trial medication is reasonable, but the event could have another, equally likely cause.
- Probably related: the relationship of the onset of the event relative to the administration of the trial medication is reasonable and the event is more likely explained by the trial medication than any other cause.
- Definitely related: the relationship of the onset of the event relative to the administration of the trial medication is reasonable and there is no other likely explanation as to the cause of the event. Where appropriate, a re-challenge with the trial medication produces another definitely related outcome.

Expected AEs: There is a small increase in the risk of autoimmune disease (notably rheumatological conditions) but this seems to be in those with established family history of those diseases; bone pain particularly hip girdle pain occurs; the previously cited risk of splenomegaly and splenic rupture seems not to be proven and patients are no longer warned of this risk (British Bone Marrow registry guidelines).

Appendix II Standard Operating Procedures

__Standard Operating Procedure__

Work-up Procedure for Trial Entry

1. Indications for Practice

1.1 Participants in the GCSF for Friedreich Ataxia Study

2. Authorised Personnel/Training Required

2.1 Trial clinicians and consultants

3. Procedure

3.1 Lead neurologist (Dr Wilkins) will have approached participant who will have indicated that they are interested in taking part in the above study. A patient information sheet will have been provided at an earlier date.

3.2 The data collection sheet 'Work-up Procedure at Trial Entry' should be completed.

3.3 The purpose of the study will be outlined again and the participant will be asked to confirm they have received a trial information sheet. The participant will be consented for entry into the study if this has not already been done.

3.4 The participant's history will be reviewed to ensure they meet the entry criteria.

Inclusion criteria:

- Genetic diagnosis of FRDA
- Age of 18 and over

Exclusion criteria:

- pregnancy, breastfeeding or lactation
- Significant abnormalities on baseline bloods (full blood count, renal and liver function)
- Previous diagnosis of haematological disorder (including malignancy)
- Previous history of splenomegaly
- Previous history of autoimmune disease

3.5 The participant will undergo a general clinical examination including a detailed neurological assessment.

3.6 The participant will be consented for blood testing and blood will be taken for U+Es, LFTs, FBC.

Standard Operating Procedure

Venesection

1. Indications for Practice

1.2 Collection of blood for analysis of the effects of GCSF on frataxin.

2. Authorised Personnel/Training Required

2.1 Any nurse or doctor who has venepuncture competency and who has been assessed as competent according to AHU guidelines. NHS Blood and Transplant staff

3. Procedure

3.1 Equipment

The following equipment is required for the procedure:

blood bottles and forms
disposable gloves
alcohol wipe
tape
cotton wool/gauze

3.2 Procedure

1. Explain the procedure to the patient before commencing to ensure the patient is aware of forthcoming actions.
2. Lie the patient down in a semi-prone position supported with pillows on a bed/reclining chair.
3. Wash hands and put on disposable gloves.
4. Identify a suitable vein for venepuncture, preferably the anti-cubital fossa.
5. Cannulate vein with needle and take blood samples.
6. Remove the needle from the vein, covering the puncture site with cotton wool or gauze and ask the patient to maintain pressure on the site to ensure the bleeding stops. Cover with tape or a plaster.
7. Record action taken in the medical notes and on trial paperwork.
8. Ensure next trial appointment has been made.
9. Discharge the patient.
