# Supplementary material for: An open-label pilot study of recombinant granulocyte-colony stimulating factor in Friedreich’s ataxia
Source: Nat Commun. 2022 Aug 9;13:4655. doi: 10.1038/s41467-022-31450-w (PMC9363409; doi:10.1038/s41467-022-31450-w)
Supplement: Supplementary file 3 — Reporting summary [file 41467_2022_31450_MOESM3_ESM.pdf]

nature research

Corresponding author(s): Alastair Wilkins

Last updated by author(s): Sep 10, 2020

Reporting Summary

Nature Research wishes to improve the reproducibility of the work that we publish. This form provides structure for consistency and transparency in reporting. For further information on Nature Research policies, see our Editorial Policies and the Editorial Policy Checklist.

Statistics

For all statistical analyses, confirm that the following items are present in the figure legend, table legend, main text, or Methods section.

n/a	Confirmed	
☐	☒	The exact sample size (*n*) for each experimental group/condition, given as a discrete number and unit of measurement
☐	☒	A statement on whether measurements were taken from distinct samples or whether the same sample was measured repeatedly
☐	☒	The statistical test(s) used AND whether they are one- or two-sided<br>*Only common tests should be described solely by name; describe more complex techniques in the Methods section.*
☒	☐	A description of all covariates tested
☐	☒	A description of any assumptions or corrections, such as tests of normality and adjustment for multiple comparisons
☐	☒	A full description of the statistical parameters including central tendency (e.g. means) or other basic estimates (e.g. regression coefficient) AND variation (e.g. standard deviation) or associated estimates of uncertainty (e.g. confidence intervals)
☐	☒	For null hypothesis testing, the test statistic (e.g. *F*, *t*, *r*) with confidence intervals, effect sizes, degrees of freedom and *P* value noted<br>*Give P values as exact values whenever suitable.*
☒	☐	For Bayesian analysis, information on the choice of priors and Markov chain Monte Carlo settings
☒	☐	For hierarchical and complex designs, identification of the appropriate level for tests and full reporting of outcomes
☒	☐	Estimates of effect sizes (e.g. Cohen's *d*, Pearson's *r*), indicating how they were calculated

Our web collection on statistics for biologists contains articles on many of the points above.

Software and code

Policy information about availability of computer code

Data collection	ImageJ version 2.0.0-rc-43/1.52n (2015), NIH.<br>Image Lab™ version 6.0.1 (2017), Bio-Rad laboratories.<br>BD FACSDiva v8.0.1 software, BD Biosciences.<br>OPTIMA software version 2.20R2, BMG Labtech.
Data analysis	GraphPad Prism version 8.4.1 for macOS (2020), GraphPad Software, USA;

For manuscripts utilizing custom algorithms or software that are central to the research but not yet described in published literature, software must be made available to editors and reviewers. We strongly encourage code deposition in a community repository (e.g. GitHub). See the Nature Research guidelines for submitting code & software for further information.

Data

Policy information about availability of data

All manuscripts must include a data availability statement. This statement should provide the following information, where applicable:

- Accession codes, unique identifiers, or web links for publicly available datasets
- A list of figures that have associated raw data
- A description of any restrictions on data availability

The authors declare that all data supporting the findings of this study are available within the paper and its supplementary information. Raw data used to calculate deviations from baseline values (Figs. 3, 4 and 5) are included in the Source data file. The study protocol is provided with this paper as Supplementary Methods. Source data are provided with this paper.

Field-specific reporting

Please select the one below that is the best fit for your research. If you are not sure, read the appropriate sections before making your selection.

- [x] Life sciences
- [ ] Behavioural & social sciences
- [ ] Ecological, evolutionary & environmental sciences

For a reference copy of the document with all sections, see nature.com/documents/nr-reporting-summary-flat.pdf

Life sciences study design

All studies must disclose on these points even when the disclosure is negative.

Sample size	No sample size calculations were performed. This was a pilot study of seven patients prior to larger phase 2/3 studies. Sample sizes were designed to characterise the outcome measures (including standard deviation required for power calculations in future studies) and based on incidence of disease in the population, willingness of patients to consider recruitment, adherence rates and time required for data collection. The sample size used in this study was sufficient to generate data to demonstrate significant changes in several outcome measures, including the primary outcome measure (changes in frataxin levels).
Data exclusions	One participant requested to be withdrawn from the trial following the first dose of G-CSF (day 1). Data relating to this participant were not included as the course of G-CSF treatment was not completed and no biological samples or data were provided post-treatment, thus the effect of treatment on all outcome measures could not be analyzed. The data exclusions reported were established before analysis of study data.
Replication	Reproducibility of the experimental findings were verified through measurements being taken from independent samples/biological replicates provided by the six study participants at multiple time points. Replication across independent samples was successful.<br>Primary outcome results (increased frataxin levels in peripheral blood mononuclear cells) were also successfully replicated in an additional peripheral blood cell population (platelets).
Randomization	This was an open-label single arm clinical trial, with all patients receiving the same intervention (G-CSF treatment) - randomized allocation of participants to treatment group was therefore not relevant to this study.
Blinding	This was an open-label single arm clinical trial, with all patients receiving the same intervention (G-CSF treatment) - blinding investigators to treatment group allocation was therefore not relevant to this study.

Reporting for specific materials, systems and methods

We require information from authors about some types of materials, experimental systems and methods used in many studies. Here, indicate whether each material, system or method listed is relevant to your study. If you are not sure if a list item applies to your research, read the appropriate section before selecting a response.

Materials & experimental systems

n/a	Involved in the study	
[ ]	[x]	Antibodies
[x]	[ ]	Eukaryotic cell lines
[x]	[ ]	Palaeontology and archaeology
[x]	[ ]	Animals and other organisms
[ ]	[x]	Human research participants
[ ]	[x]	Clinical data
[x]	[ ]	Dual use research of concern

Methods

n/a	Involved in the study	
[x]	[ ]	ChIP-seq
[ ]	[x]	Flow cytometry
[x]	[ ]	MRI-based neuroimaging

Antibodies

Antibodies used	anti-CD45-PE (555483; Clone HI30; BD Biosciences)<br>anti-CD34-FITC (555821; Clone 581; BD Biosciences)<br>anti-CD133-APC (130-113-106; Clone AC133; Miltenyi Biotec)<br>anti-beta actin (ab8227; Abcam)<br>anti-NRF1 (ab175932; Clone EPR5554(N); Abcam)<br>anti-Nrf2 (sc-722; Clone C-20; Santa Cruz Biotechnology)<br>anti-PGC-1α (sc-13067; Clone H-300; Santa Cruz Biotechnology)<br>horseradish peroxidase–conjugated goat anti-rabbit IgG (ab6721; Abcam)
Validation	Antibodies were validated by both the manufacturers and our previous studies:<br>1. Redondo J, et al. Dysregulation of mesenchymal stromal cell antioxidant responses in progressive multiple sclerosis. Stem Cells Transl Med 7, 748-758 (2018).

2. Kemp KC, et al. Bone marrow transplantation stimulates neural repair in Friedreich's ataxia mice. Ann Neurol 83, 779-793 (2018).
3. Kemp KC, et al. Cytokine therapy-mediated neuroprotection in a Friedreich's ataxia mouse model. Ann Neurol 81, 212-226 (2017).
4. Kemp K et al. Mesenchymal Stem Cell-Derived Factors Restore Function to Human Frataxin-Deficient Cells. Cerebellum 16, 840-851 (2017).

Human research participants

Policy information about studies involving human research participants

Population characteristics	Seven participants with genetically confirmed diagnosis of Friedreich's ataxia (GAA-repeat expansion on both alleles of the FXN gene) were recruited to the study. A detailed overview of the participant covariate-relevant population characteristics are provided within the paper (Table 1).
Recruitment	Participants were recruited via Neurology Clinics at North Bristol NHS Trust (Southmead Hospital, Bristol, UK). Male and female participants aged over 18 years were eligible to participate if they had a genetically confirmed diagnosis of FA (GAA-repeat expansion on both alleles of the FXN gene). Exclusion criteria included participation in other clinical trials within 30 days of the initial dose of G-CSF; pregnancy, breastfeeding or lactation; current or previous diagnosis of serious medical disorders or illnesses including haematologic disease (including malignancy), splenomegaly, autoimmune disease, pulmonary infiltrate, pulmonary fibrosis or haemoptysis; and clinical abnormalities on baseline bloods (full blood count [FBC], renal and liver function). No self-selection bias is anticipated
Ethics oversight	East of England - Cambridge East Research Ethics Committee (REC 17/EE/0486), UK

Note that full information on the approval of the study protocol must also be provided in the manuscript.

Clinical data

Policy information about clinical studies

All manuscripts should comply with the ICMJEguidelines for publication of clinical research and a completedCONSORT checklist must be included with all submissions.

Clinical trial registration	EudraCT 2017-003084-34
Study protocol	The study protocol is provided as Supplementary information.
Data collection	Southmead Hospital, Bristol UK; Start date: 2018-01-22; Date of the global end of the trial: 2019-04-12
Outcomes	Primary outcome measure:<br>Changes in frataxin levels in peripheral blood mononuclear cells after granulocyte-colony stimulating factor (G-CSF) administration to patients with Friedreich’s Ataxia . Frataxin levels in peripheral blood mononuclear cells were assessed prior to, and for 19 days following G-CSF administration using the Frataxin Protein Quantity Dipstick Assay Kit (Abcam).<br><br>Secondary outcome measures:<br>Safety of G-CSF administration to patients with Friedreich’s Ataxia. Safety was assessed by clinical examination, reporting of adverse events and laboratory blood monitoring (full blood count, renal and liver function) prior to, and for 19 days following G-CSF administration.<br>Changes in mitochondrial enzyme activity (aconitase and succinate dehydrogenase) and/or antioxidant levels (PGC-1α, NRF1 and Nrf2) in peripheral blood mononuclear cells after G-CSF administration to patients with Friedreich’s Ataxia. Enzyme activities and antioxidant levels in peripheral blood mononuclear cells were assessed prior to, and for 19 days following G-CSF administration using colorimetric enzyme activity assays and immuno dot-blot assays respectively.

Flow Cytometry

Plots

Confirm that:

- [x] The axis labels state the marker and fluorochrome used (e.g. CD4-FITC).
- [x] The axis scales are clearly visible. Include numbers along axes only for bottom left plot of group (a 'group' is an analysis of identical markers).
- [x] All plots are contour plots with outliers or pseudocolor plots.
- [x] A numerical value for number of cells or percentage (with statistics) is provided.

Methodology

Sample preparation	Whole blood was collected into EDTA Vacutainers™ (BD Bioscience). Blood samples were incubated with antibodies anti-CD45-PE (1:5; 555483; BD Biosciences), anti-CD34-FITC (1:5; 555821; BD Biosciences) and anti-CD133-APC (1:10; 130-113-106; Miltenyi Biotec) in BD Trucount tubes (BD Biosciences) for 15mins at room temperature. Mature red blood cells were lysed with FACS lysis buffer (BD Biosciences) and samples suspended in phosphate-buffered saline.
Instrument	BD FACSCanto flow cytometer, BD Biosciences.

Software	BD FACSDiva v8.0.1 software, BD Biosciences.
Cell population abundance	At least 85,000 events per blood sample were acquired.
Gating strategy	CD34 and CD133 positive stem cells were quantified, using BD FACSDiva v8.0.1 software (BD Biosciences), based on the standardised International Society for Hematotherapy and Graft Engineering (ISHAGE) gating protocol. A figure exemplifying the gating strategy is provided in the Supplementary Information.

☒ Tick this box to confirm that a figure exemplifying the gating strategy is provided in the Supplementary Information.
